# Cost-accuracy and patient experience assessment of blood pressure monitoring methods to diagnose hypertension: A comparative effectiveness study

**DOI:** 10.3389/fmed.2022.827821

**Published:** 2022-11-10

**Authors:** Luis González-de Paz, Belchin Kostov, Xavier Freixa, Carmen Herranz, Elena Lagarda, María Ortega, Elisa Pérez, Silvia Porcar, Eva Sánchez, Montserrat Serrato, Ingrid Vidiella, Antoni Sisó-Almirall

**Affiliations:** ^1^Consorci d’Atenció Primària de Salut Barcelona Esquerra (CAPSBE), Barcelona, Spain; ^2^Primary Healthcare Transversal Research Group, Institut d’Investigacions Biomèdiques August Pi i Sunyer (IDIBAPS), Barcelona, Spain; ^3^Department of Statistics and Operational Research, Universitat Politècnica de Catalunya (UPC), Barcelona, Spain; ^4^School of Medicine and Health Sciences, University of Barcelona, Barcelona, Spain

**Keywords:** blood pressure, hypertension diagnosis, patient experience, cost analysis, primary care, cost-effectiveness research

## Abstract

**Objectives:**

Studies of the diagnosis of hypertension have emphasized long-term cost-effectiveness analysis, but the patient experience and costs of blood pressure monitoring methods at the diagnosis stage remain unclear. We studied four diagnostic methods: a new 1 h-automated office blood pressure (BP) monitoring, office BP measurement, home BP monitoring, and awake-ambulatory BP monitoring.

**Methods:**

We carried out a comparative effectiveness study of four methods of diagnosing hypertension in 500 participants with a clinical suspicion of hypertension from three primary healthcare (PHC) centers in Barcelona city (Spain). We evaluated the time required and the intrinsic and extrinsic costs of the four methods. The cost-accuracy ratio was calculated and differences between methods were assessed using ANOVA and Tukey’s honestly significant difference (HSD) *post-hoc* test. Patient experience data were transformed using Rasch analysis and re-scaled from 0 to 10.

**Results:**

Office BP measurement was the most expensive method (€156.82, 95% CI: 156.18–157.46) and 1 h-automated BP measurement the cheapest (€85.91, 95% CI: 85.59–86.23). 1 h-automated BP measurement had the best cost-accuracy ratio (€ 1.19) and office BP measurement the worst (€ 2.34). Home BP monitoring (8.01, 95% CI: 7.70–8.22), and 1 h-automated BP measurement (7.99, 95% CI: 7.80–8.18) had the greatest patient approval: 66.94% of participants would recommend 1 h-automated BP measurement as the first or second option.

**Conclusion:**

The relationship between the cost-accuracy ratio and the patient experience suggests physicians could use the new 1 h-automated BP measurement as the first option and awake-ambulatory BP monitoring in complicated cases and cease diagnosing hypertension using office BP measurement.

## Introduction

The diagnosis of hypertension requires accurate blood pressure (BP) monitoring (1). Currently, international guidelines accept four methods: the reference 24-h ambulatory blood pressure monitoring (24 h-ABPM), home blood pressure monitoring (HBPM), office blood pressure measurement (OBPM), and automated office blood pressure measurement (AOBP) (2–4).

In daily clinical practice, 24 h-ABPM is not feasible in all cases due to the cost, the limited availability of devices, and possible patient distress. Therefore, HBPM or OBPM is often used to diagnose hypertension (5, 6). OBPM results in a considerable proportion of false positive and false negative diagnoses in patients with white coat syndrome or masked hypertension. HBPM is a good alternative, although most patients do not have a device at home before the diagnosis and carry out BP readings in unsuitable conditions (7). Automated BP devices, such as AOBP, are considered unattended office BP monitoring (8). AOBP reduces the white coat effect and measurements are close to mean daytime pressures obtained with ABPM or HBPM. Physicians accept AOBP because it offers measurements in a relatively controlled environment, and the results are obtained rapidly (9). At present, the Canadian hypertension guidelines and the American Heart Association accept AOBP as a method of diagnosing hypertension (10, 11). However, AOBP is not uniform (12): the first described AOBP method recommended three to four BP readings over 5 min, while others suggest more (13).

A recent paper reported on 1-h Automated Office Blood Pressure (1 h-AOBP) measurement consisting of BP measurement for 1 h every 5 min using the 24-h ABPM device. 1 h-AOBP has a sensitivity of 76.6% (95% CI: 71.1–81.5), a specificity of 64.8% (95% CI: 57–72.1), with a diagnostic accuracy greater than OBP and HBPM (14). 1 h-AOBP showed similar results to HBPM and a reasonable variability compared with awake-ABPM. ABPM is the gold standard; however, it requires a device for the patient for 24 h, and the high incidence of hypertension does not allow all patients to undergo screening with this method (15). HBPM at home or the pharmacy is a good alternative; however, clinicians often suspect patients’ technique is incorrect (2). OBPM has been reported to result in overdiagnosis in 15–30% of cases and a higher risk of white coat hypertension (5). Therefore, 1 h-AOBP, which uses the same devices as ABPM for 1 h, allows multiple daily screenings and avoids common disadvantages.

Physician decision-making when choosing a hypertension diagnosis method is likely to vary during hypertension follow-up (16). In primary healthcare (PHC) clinical practice or outpatient clinics, where patients are highly independent and the hypertension diagnosis is opportunistic when exploring risk factors, physician choices are mostly discretional. Family physicians and PHC nurses might prefer BP measurement methods according to individual patient choices when they are unable or unwilling to carry out a specific BP measurement method (17). However, there is a lack of evidence on patient preferences at the time of the diagnosis of hypertension. Physician decision-making in the choice of hypertension methods is also hampered by the lack of evidence on the direct costs because studies have focused on long-term cost-effectiveness (18, 19). Therefore, the objective of this study was to evaluate the factors that most influence physicians and patients when choosing a BP measurement method: the accuracy of the method, the costs, and the patient experience. We conducted a comparative effectiveness study to evaluate four hypertension diagnostic methods, a 1-h automated office blood pressure (1 h-AOBP), awake-ABPM, HBPM, and OBPM.

## Materials and methods

### Study context

We designed a comparative effectiveness study using the view of the National Academies of Science to examine the cost-accuracy and patient experience outcomes of the hypertension diagnosis (20). This comparative effectiveness study was part of a clinical trial carried out in three PHC centers in Barcelona (Spain). The results of the clinical trial are published (14), and details of the study, the patient sample, and the characteristics of the protocol are described elsewhere (21).

The four BP methods are described elsewhere (22), and the BP methods studied were carried out according to clinical guideline recommendations (12). Participants underwent 24 h-ABPM, 1 h-AOBP, HBPM, and OBPM. 1 h-AOBP used a 24-h ABPM device set up to measure BP every 5 min for 1 h. During the hour, the patient remained in the waiting room or in a quiet consulting room in the PHC center, without walking actively, eating, or smoking. After 1 h, the device and arm cuff were removed. HBPM was carried out according to daily clinical practice: Participants were required to measure three BP readings over 3 days, but if they did not have a valid BP device at home, they were asked to record the BP in the pharmacy following the same requirements as at home. A summary of the characteristics of BP methods and requirements are in Supplementary Table 1 and all BP diagnostic methods are described.

A total of 500 patients referred by family physicians for a hypertension diagnosis underwent the four BP methods. Exclusion criteria were severe physical or cognitive limitations, episodes of any arrhythmia, any disease-causing permanent tremor, arm circumference > 42 cm, arterial-venous fistula in the arm, mental disorders or intolerance to the BP measurement method, inability to attend the study at the PHC or programmed hospitalization during the study. Data collection commenced in June 2017 and the completion date was December 2019.

### Data collection and outcomes

The time horizon of the study was determined as the time when the family physician or nurse required a BP test for hypertension to the obtention of the results. After each BP method, participants completed a questionnaire on their experience. This included comfort, time the test required, and the degree of recommendation. Five-point visual scales were used to assess comfort and time with higher points indicating better patient experience. The degree of recommendation was an item to rank the order of the four BP methods if patients had to recommend them to other patients. The patient experience data collection sheet is reported elsewhere, and further details of the data collection are published and public available (21). Accuracy figures were used as reported, using Awake-ABPM as the reference method, with a diagnostic accuracy of 100% (14). Accuracy reflected the probability that an individual would be correctly classified by the method.

The cost of the visit was calculated according to Catalan public healthcare system costs (23). Expenses reported by participants (if any) and the cost of travel to the PHC were calculated. In all cases, we considered a round trip for each visit. Public transport (metro and bus) costs were estimated (24). Taxi costs were calculated according to the price per minute, established by the Barcelona metropolitan area as € 0.56 per minute (25), and the cost of traveling in private cars was estimated per minute according to the scheme of allocation for travel expense of the Spanish Tax Agency (26). The cost-accuracy ratio estimated how much it cost to gain a unit of diagnostic accuracy. Additionally, the time was estimated in minutes as the time used to reach the PHC reported by participants added to an estimated time of 10 min per consultation. In the case of 1 h-AOBP we added 60 min because the test takes place during this time in the PHC.

### Statistical analysis

We carried out a cost-accuracy study with a similar perspective to a cost-effectiveness analysis (27). Categorical variables were presented as absolute frequencies and percentages and continuous variables as mean and standard deviation (SD). The time necessary was calculated by adding the transport times and consultation times per patient; costs were calculated by adding the consultation rates, transportation costs, and patient expenses. The patient experience results from the questionnaire were transformed into interval scaling using a Rasch analysis (28), which first required verification of the goodness-of-fit of the data to the model. Patient experience logit units from the Rasch analysis were re-scaled to 0–10, where 10 meant the best comfort and time experience and 0 the worst experience. Patient experiences of BP methods were studied by examining the overall mean experience and patient characteristics using ANOVA. We used sociodemographic variables to examine between-group differences: age, sex, educational level, and ethnicity. We analyzed the proportion of the rank order of the four BP methods with charts where the participant had to recommend each BP method to other patients.

The cost study was carried out using ANOVA tests with the mean costs and time of each BP method. In all analyses, the results were expressed as mean and 95% confidence intervals (95% CI). Differences between BP methods were examined using Tukey’s honestly significant difference (HSD) post hoc test. The analysis was made using Winsteps Rasch software and R version 3.6.0 for Windows (29, 30).

### Ethics

The study was approved by the Ethics Committee of the Hospital Clinic of Barcelona (ref. number HCB/2014/0615) and registered at ClinicalTrials.gov (NCT03147573). All participants were informed of the study aims and provided written informed consent.

## Results

Of the 500 patients included, 244 (48.8%) were female, with a mean age of 59.7 years (SD 14.1 years). Most participants had a tertiary education and reached the PHC center by walking < 13 min; they were not diagnosed with hypertension but 53.8% of participants reported having a sphygmomanometer at home. Participant characteristics are shown in Table 1.

**TABLE 1:** Participant characteristics.

Characteristics	*N* = 500
**Sex, female**	244 (48.8)
**Age (years)**	59.7 ± 14.1
**Ethnicity**	
Caucasian	434 (86.8)
Latin	53 (10.6)
Other	13 (2.6)
**Educational level**	
Without studies	15 (3.0)
Primary school	56 (11.2)
High-school (up to 16 years)	71 (14.2)
High school or vocational studies (up to 18 years)	129 (25.8)
University studies or higher	229 (45.8)
**Time used to reach the primary health care facility (minutes)**	**12.3 ± 8.9**
**Transport used**	
Walking	430 (86.0)
Private car	30 (6.0)
Public transport (metro, bus)	36 (7.2)
Taxi	4 (0.8)
**Had a sphygmomanometer at home**	**269 (53.8)**
**Went to a pharmacy to check blood pressure (HBPM)**	**242 (48.4)**
**Mean price paid at pharmacy**	**€0.6 ± 1.3**

Data are presented as *n* (%) or mean ± standard deviation.

The costs, diagnostic accuracy, and time study in Table 2 shows that 1 h-AOBP was the cheapest (€85.91, 95% CI: €85.59–€86.23), while OBPM was the most expensive with a mean cost of €156.82.91 (95% CI: €156.18–€157.46). On average, HBPM required a shorter time to diagnosis than the other methods. The cost-accuracy ratio showed that HBPM, 1 h-AOBP and awake-ABPM were very similar, while the OBPM had an almost twofold higher cost-accuracy ratio.

**TABLE 2:** Cost and time study of diagnosing hypertension.

Method	Total transport costs [95% CI]	Time necessary in minutes (consultations and transport) [95% CI]	Total cost [95% CI]	Cost-accuracy ratio [95% CI]
Awake-ABPM	€ 1.37 [0.89–1.85]	103.33 [98.60–108.06]	€ 121.38 [120.89–121.85]	1.21 [1.24–1.25]
OBPM.	€ 1.82 [1.18–2.46]	137.78 [131.46–144.09]	€ 156.82 [156.18–157.46]	2.34 [2.33–2.34]
HBPM	€ 0.91 [0.59–1.23]	68.88 [65.73–72.04]	€ 86.23 [85.90–86.56]	1.25 [1.24–1.25]
1 h-AOBP.	€ 0.91 [0.59–1.23]	118.88 [115.73–122.04]	€ 85.91 [85.59–86.23]	1.19 [1.24–1.25]

Data are presented as mean [95% confidence intervals]. Awake-ABPM, Awake ambulatory blood pressure monitoring; OBPM, Office blood pressure measurement; HBPM, Home blood pressure monitoring; 1 h-AOBP, 1 h- automated office blood pressure measurement.

According to patient experience, the highest rated BP methods were HBPM and 1 h-AOBP with 8 out of 10, and the least valued was awake-ABPM with a mean score of 5.19 (95% CI: 4.98–5.40). Pairwise comparison analysis showed greater differences (> 2 points) in comparisons of methods with awake-ABPM. The results are shown in Table 3. ANOVA tests of the mean experience by BP method and group characteristics showed significant but not relevant differences (<1 point) by age group in OBP, HBPM, and 1 h-AOBP. The results of this analysis are shown in Supplementary Table 2.

**TABLE 3:** ANOVA test and pairwise comparisons (Tukey HSD tests) to examine the patient experience of each BT method.

BP test	Patient experience (handling and time 0–10 scale) mean and [95% CI]	*F*	*P*-value
Awake-ABPM	5.19 [4.98–5.40]	151.8	<0.001
OBPM.	7.32 [7.55–7.09]		
HBPM.	8.01 [7.70–8.22]		
1 h-AOBP.	7.99 [7.80–8.18]		

**Pairwise comparisons**	**Mean difference and 95% CI**	***P*-value**	

OBPM vs. awake-ABPM	2.14 [1.74–2.53]	<0.001	
1 h-AOBP vs. awake-ABPM	2.80 [2.41–3.19]	<0.001	
1 h-AOBP vs. OBPM	0.67 [0.27–1.06]	<0.001	
HBPM vs. awake-ABPM	2.82 [2.43–3.22]	<0.001	
HBPM vs. OBPM	0.68 [0.29–1.08]	<0.001	
HBPM vs. 1 h-AOBP	0.02 [−0.38 to 0.41]	0.999	

**FIGURE 1:**
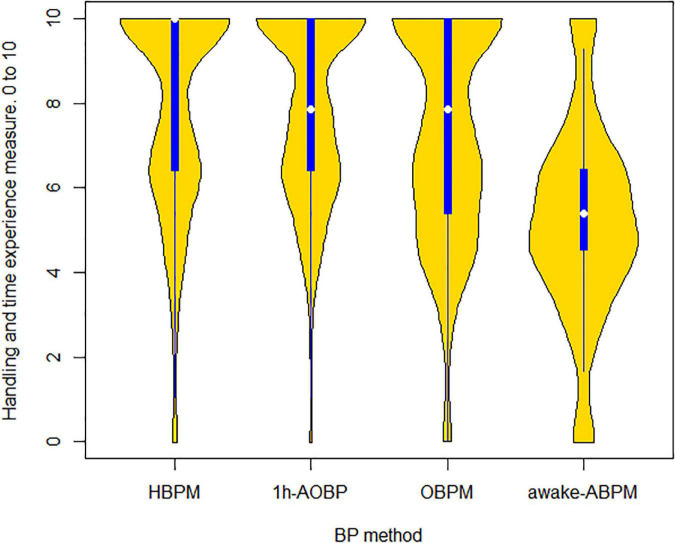
Distribution plot with the experience (0–10 scale) by BP method. The yellow shape shows density of the data at different values of patient experience, and the blue line and white dot are the inter-quartile rank and median, respectively.

**FIGURE 2:**
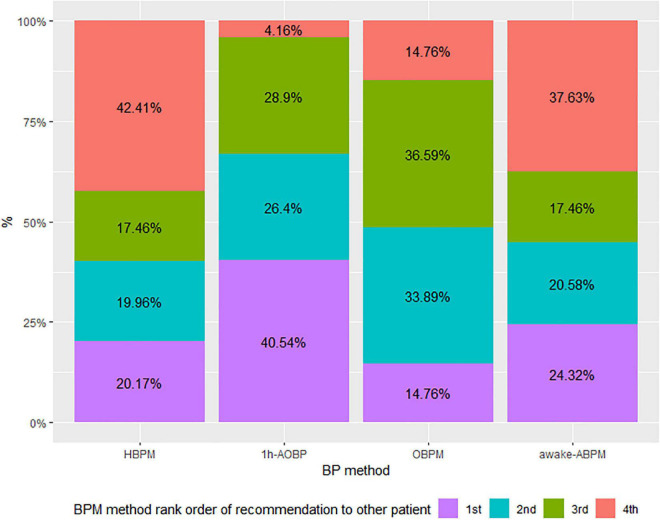
Lower part: staked bar chart with participants rank order of recommendation of each BP method to other patients. HBPM, Home blood pressure monitoring. 1 h-AOBP, 1 h- automated office blood pressure measurement; OBPM, Office Blood Pressure measurement; Awake-ABPM, Awake ambulatory blood pressure monitoring.

**FIGURE 3:**
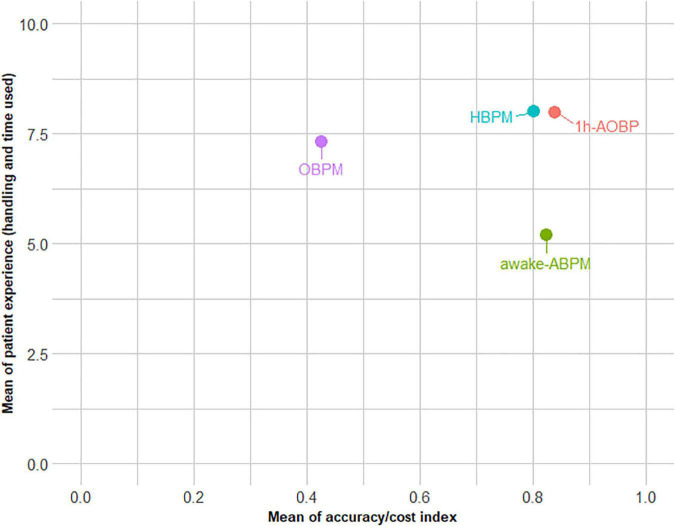
Relationship between the cost-accuracy ratio and the patient experience. HBPM, Home blood pressure monitoring. 1 h-AOBP, 1 h- automated office blood pressure measurement; OBPM, Office Blood Pressure measurement; Awake-ABPM, Awake ambulatory blood pressure monitoring.

Figure 1 shows the differences in the distribution of the patient experience by BP method. The HBPM and 1 h-AOBP produced almost the same experience, while the worst rated was awake-ABPM. The stacked bar chart in the Figure 2 shows that 66.94% of participants would recommend 1 h-AOBP to other patients as the first or second option. Figure 3 shows the relationship between the cost-accuracy ratio, and the patient experience. BP methods with better cost-accuracy figures (close to 1 in the x axis) were 1 h-AOBP and HBPM, with a similar ratio to the gold-standard of awake-ABPM. However, 1 h-AOBPM and HBPM showed the best relationship according to the cost-accuracy ratio and high patient experience (y axis).

## Discussion

We evaluated four methods of diagnosing hypertension according to cost-accuracy and patient experience. The results showed 1 h-AOBP had the best cost-accuracy, and the patient experience was similar to HBPM. 1 h-AOBP and HBPM showed the best outcome indicators compared with OBPM and awake-ABPM.

### Patient experience

Patient preferences for handling and the time required was clearly favorable to 1 h-AOBP and HBPM. The lower patient endorsement of OBPM might be influenced by the number of consultations and the time required (30). In contrast, awake-ABPM, although it only requires two consultations, has the drawback of wearing the BP measurement cuff and device for 1 day, meaning patients cannot carry out their daily activities (e.g., ride a motorcycle or any activities involving physical effort) (31). Patients clearly endorsed 1 h-AOBP and HBPM over other BP methods, and this was not associated with patient characteristics. Therefore, 1 h-AOBP and HBPM may be the best methods for physicians based on the patient experience. Studies of patient experiences in the diagnosis of hypertension show that HBPM is better accepted than OBPM, mainly because of patient confidence in the accuracy of BP measurement (32). However, a nationwide physician survey in Canada—where AOBP is most established, since the Canadian Hypertension guidelines included it as a valid method—showed that the preferred method of hypertension screening remains OBPM carried out with mercury devices (54.2%) compared with AOBP (38.8%) (33). A similar trend was observed in a survey carried out in a large sample of physicians in Hong Kong with a lower proportion of AOBP use (13.7%) and no use of HBPM (34). In Europe, a recent BP monitoring and measurement method guideline incorporated AOBP, although with relatively little information on it (35). While OBPM has the worst indicators and HBPM at the diagnostic stage might cause suspicion in clinicians, we suggest that our results might persuade healthcare providers and physicians to opt for new AOBP methods when selecting the best monitoring to diagnose hypertension.

### Cost of diagnosing hypertension

The cost study showed 1 h-AOBP had results similar to HBPM, and the most expensive method was OBPM. Likewise, the cost-accuracy analysis showed OBPM was almost twice as good as the other methods, while 1 h-AOBP and awake-ABPM had the best cost-accuracy values. Some reports advise AOBP in all patients due to time or cost factors (31). Our results show that AOBP is cheaper than other diagnostic methods. To our knowledge, this approach to the effectiveness of BP measurement methods has not been studied, as researchers have prioritized cost-effectiveness (18). The evaluation of diagnostic methods often stops after quantifying the accuracy of the method compared with the gold-standard test (36). However, in the diagnosis of hypertension, BP measurement is linked to the patient experience and direct and indirect patient costs (37), and, therefore, incorporating the patient experience within the cost-accuracy framework is relevant in the evaluation of replacing OBPM by 1 h-AOBP in daily clinical practice: 1 h-AOBP favors the three domains (cost, patient outcomes and diagnostic accuracy).

The difference between the most expensive and the cheapest BP method (OBPM vs. 1 h-AOBP) was €-70.91. These results reflect real costs and have not been inflated but emerged from the reality of the well-established Spanish PHC system. In Spain, PHC is a core element of the health system, and is provided by specialist family physicians and nurses (38). PHC is managed, with few exemptions, by public trusts funded from the public budget according to block grants, including acute and chronic care, health promotion and prevention activities (39). While the benefits are not subject to patient cost-sharing, our results show that patients share indirect costs (e.g., number of visits). The results on effectiveness, in terms of real costs, of a PHC system according to the case study of diagnosing hypertension with different BP methods meant we adapted the number of visits to daily clinical practice differences in reimbursements, which might be higher but might not alter the ranking of costs of BP methods. We propose a feasible strategy to use 1 h-AOBP according to the results and the sensibility and specificity of 1 h-AOBP (12): (1) If hypertension is suspected—not restricted to opportunistic screening in the office—the physician should program 1 h-AOBP, asking the patient to bring the HBPM results. (2) Once the physician has the 1 h-AOBP and HBPM results they will decide whether hypertension can be diagnosed or, (3) in case of doubt, program 24 h-ABPM as a confirmatory method. We suggest this strategy could deal with some of the problems derived from false negatives and false positives and reduce the difference in cost-utility with awake or 24 h-ABPM (15).

### Limitations and strengths of the study

The study has some limitations. We carried out a cost-accuracy and patient experience study to diagnose hypertension, but not the follow up, and only studied direct costs. A health technology assessment modeling study might answer the question of the indirect costs, but this was not the aim of our study. We aimed to motivate implementation of the most cost-accurate BP method to diagnose hypertension and suggest this is the first step to plan a cost-effectiveness study of 1 h-AOBPM. However, there is abundant literature on the follow up of hypertension, and the diagnostic stage is critical for subsequent monitoring. The reference test to diagnose hypertension is the 24 h-ABPM has limitations with the availability of the devices. However, this study examined the cost-accuracy and patient experience indicators of the four most important BP methods to diagnose hypertension. The results provide evidence of all advantages and drawbacks to help clinicians make better-informed decisions. The associated costs correspond to PHC and may seem low. However, we suggest the relative differences between BP methods can be adapted to other healthcare provision, including hospitals. Although the diagnosis of hypertension is highly dependent on the physician, our results come from real-life conditions and therefore physicians and healthcare managers could use the results to establish standardized procedures.

## Conclusion

We found 1 h-AOBP had the best cost-accuracy ratio, with patient time and comfort indicators similar to HBPM. The 1 h-AOBP indicators of cost-accuracy and patient experience might impact the speed of the diagnosis. Our findings are important with respect to clinical strategies, as OBPM as a diagnostic strategy for hypertension in PHC could cease, as it has the highest costs, the lowest accuracy and is not well accepted by patients. 1 h-AOBP might reduce healthcare diagnostic costs and improve patients’ healthcare experience.

## Data availability statement

The raw data supporting the conclusions of this article will available by the authors upon reasonable request.

## Ethics statement

The studies involving human participants were reviewed and approved by the Hospital Clinic of Barcelona (Spain). The patients/participants provided their written informed consent to participate in this study.

## Author contributions

LG-dP, BK, and AS-A: conceptualization, methodology, and writing—review and editing. BK: formal analysis. XF, CH, EL, MO, EP, SP, ES, MS, IV, LG-dP, and BK: resources and data curation. LG-dP: writing—original draft preparation. LG-dP and AS-A: funding acquisition. All authors have read and agreed to the published version of the manuscript.
