# Supplementary material for: Cost-accuracy and patient experience assessment of blood pressure monitoring methods to diagnose hypertension: A comparative effectiveness study
Source: Front Med (Lausanne). 2022 Nov 10;9:827821. doi: 10.3389/fmed.2022.827821 (PMC9684318; doi:10.3389/fmed.2022.827821)
Supplement: Supplementary file 1 [file Table_1.pdf]

Supplementary Material

Method.	Hypertension threshold (mmHg)	Number of BP measurements	Sensitivity (%)	Specificity (%)	Diagnostic accuracy (%)	Consultations needed.		Total cost (consults)	Number of round trips to the PHC center
Awake-ABPM	SBP≥135, & or DBP≥85	49 to 52	*Ref*	*Ref*	100	PHC Nurse<br>GP	2<br>1	€120	6
OBPM	SBP≥140 SBP, & or DBP≥90	3	76.9	50.3	66.9	PHC Nurse<br>GP	3<br>1	€155	8
HBPM	SBP≥135 SBP, & or DBP≥85	3	81.7	48.5	69.2	PHC Nurse<br>GP	1<br>1	€85	4
1h-AOBP	SBP≥135 SBP, & or DBP≥85	13	76.6	64.8	72.1	PHC Nurse<br>GP	1<br>1	€85	4

Supplementary Table I: Characteristics of BP methods.
SBP: Systolic blood pressure, DBP: diastolic blood pressure
GP: General Practitioner.
Awake-ABPM: Awake ambulatory blood pressure monitoring
OBPM: Office blood pressure measurement.
HBPM: Home blood pressure monitoring.
1h-AOBP: 1h-automated office blood pressure measurement.

BP test	Characteristic	Group	Handling and time experience. Mean (SD)	*F*	P-value
**24h-ABPM Day-time.**	**Sex**	Males	5.25 (2.39)	0.405	0.525
		Females	5.12 (2.37)		
	**Age (years)**	[23.56 to 49.86)	5.01 (2.31)	2.346	0.126
		[49.86 to 60.59)	4.94 (2.52)		
		[60.59 to 70.17)	5.5 (2.45)		
		[70.17 to 101.43]	5.32 (2.2)		
	**Ethnicity**	Caucasian	5.21 (2.37)	0.172	0.842
		Latin	5.05 (2.51)		
		Other	4.95 (2.44)		
	**Academic level**	Without studies	5.12 (2.33)	0.282	0.89
		Primary school	5.43 (2.64)		
		High-school (up to 16 years)	5.33 (2.66)		
		High school (up to 18 years)	5.12 (2.43)		
		University studies or higher	5.12 (2.21)		
**OBPM.**	**Sex**	Males	7.11 (2.6)	3.408	0.065
		Females	7.54 (2.61)		
	**Age (years)**	[23.56 to 49.86)	6.74 (2.69)	7.898	*0.005*
		[49.86 to 60.59)	7.34 (2.73)		
		[60.59 to 70.17)	7.47 (2.45)		
		(70.17 to 101.43]	7.72 (2.47)		
	**Ethnicity**	Caucasian	7.38 (2.61)	0.789	0.455
		Latin	7.02 (2.55)		
		Other	6.73 (2.79)		
	**Academic level**	Without studies	7.5 (3.31)	0.422	0.793
		Primary schools	7.49 (2.42)		
		High-school (up to 16 years)	7.21 (2.87)		
		High school (up to 18 years)	7.52 (2.34)		
		University studies or higher	7.19 (2.68)		
**HBPM.**	**Sex**	Males	7.93 (2.39)	0.576	0.448
		Females	8.09 (2.49)		
	**Age (years)**	[23.56 to 49.86)	7.4 (2.62)	8.196	*0.004*
		[49.86 to 60.59)	8.04 (2.42)		
		[60.59 to 70.17)	8.16 (2.42)		
		[70.17 to 101.43]	8.35 (2.25)		

BP test	Characteristic	Group	Handling and time experience. Mean (SD)	*F*	P-value
HBPM	Ethnicity	Caucasian	8.05 (2.4)	0.627	0.534
		Latin	7.83 (2.67)		
		Other	7.38 (2.9)		
	Academic level	Without studies	8.21 (2.62)	1.93	0.104
		Primary schools	8.11 (2.55)		
		High-school (up to 16 years)	8.73 (1.71)		
		High school (up to 18 years)	7.84 (2.52)		
		University studies or higher	7.85 (2.52)		
AOBPM-1h.	Sex	Males	7.99 (2.18)	0	0.994
		Females	7.99 (2.17)		
	Age (years)	[23.56 to 49.86)	7.64 (2.42)	4.775	*0.029*
		[49.86 to 60.59)	7.7 (2.14)		
		[60.59 to 70.17)	8.61 (1.92)		
		[70.17 to 101.43]	7.99 (2.12)		
	Ethnicity	Caucasian	8 (2.12)	0.732	0.482
		Latin	8.12 (2.36)		
		Other	7.31 (3.1)		
	Academic level	Without studies	8.55 (1.66)	1.691	0.151
		Primary schools	7.76 (2.23)		
		High-school (up to 16 years)	8.54 (2.04)		
		High school (up to 18 years)	7.86 (2.19)		
		University studies or higher	7.92 (2.2)		

Supplementary table 2: ANOVA test study to examine differences in the mean experience across groups.
